# John D. Chevalier

**Published:** 1851-04

**Authors:** 


*John D. Chevalier.*—We are sorry to learn that the extensive dental and surgical instrument manufactory of Mr. Chevalier, of New York, was recently consumed by fire. We trust that his loss was covered by insurance, and that he will soon be able to fill orders from the profession with his usual promptness.

